# Antisense expression of the fasciclin-like arabinogalactan protein *FLA6* gene in *Populus* inhibits expression of its homologous genes and alters stem biomechanics and cell wall composition in transgenic trees

**DOI:** 10.1093/jxb/eru479

**Published:** 2014-11-26

**Authors:** Haihai Wang, Chunmei Jiang, Cuiting Wang, Yang Yang, Lei Yang, Xiaoyan Gao, Hongxia Zhang

**Affiliations:** ^1^National Key Laboratory of Plant Molecular Genetics, Shanghai Institute of Plant Physiology and Ecology, Chinese Academy of Sciences, 300 Fenglin Road, Shanghai 200032, PR China; ^2^College of Life Sciences, Nanjing University, 22 Hankou Road, Nanjing 210093, PR China

**Keywords:** Biomechanics, cell wall, fasciclin-like arabinogalactan protein, *Populus*, transgenic plant, xylem.

## Abstract

Fasciclin-like arabinogalactan proteins (FLAs) play important roles in the growth and development of roots, stems, and seeds in *Arabidopsis*. However, their biological functions in woody plants are largely unknown. In this work, we investigated the possible function of *PtFLA6* in poplar. Quantitative real-time PCR, PtFLA6–yellow fluorescent protein (YFP) fusion protein subcellular localization, Western blotting, and immunohistochemical analyses demonstrated that the *PtFLA6* gene was expressed specifically in the xylem of mature stem, and PtFLA6 protein was distributed ubiquitous in plant cells and accumulated predominantly in stem xylem fibres. Antisense expression of *PtFLA6* in the aspen hybrid clone *Poplar davidiana*×*Poplar bolleana* reduced the transcripts of *PtFLA6* and its homologous genes. Transgenic plants that showed a significant reduction in the transcripts of *PtFLA*s accumulated fewer PtFLA6 and arabinogalactan proteins than did the non-transgenic plants, leading to reduced stem flexural strength and stiffness. Further studies revealed that the altered stem biomechanics of transgenic plants could be attributed to the decreased cellulose and lignin composition in the xylem. In addition expression of some xylem-specific genes involved in cell wall biosynthesis was downregulated in these transgenic plants. All these results suggest that engineering the expression of *PtFLA6* and its homologues could modulate stem mechanical properties by affecting cell wall composition in trees.

## Highlight

Fasciclin-like arabinogalactan proteins, a class of cell wall glycoprotein, function in maintaining stem biomechanics and cell wall composition in *Populus.*

## Introduction

As a typical class of glycoprotein, arabinogalactan proteins (AGPs) are involved in various aspects of plant growth and development, such as pollen-tube growth (Levitin *et al.*, 2008; Coimbra *et al.*, 2009), apical cell expansion (Lee *et al.*, 2005), stem development (Park *et al.*, 2003), cell division and expansion (Yang *et al.*, 2007), xylem differentiation (Motose *et al.*, 2004), and root development (van Hengel and Roberts, 2003). Fasciclin-like AGPs (FLAs) are a subclass of AGPs that contain one or two fasciclin domains similar to the fasciclin cell-adhesion molecules in *Drosophila melanogaster* (Gaspar *et al.*, 2001). In *Arabidopsis*, based on the number of fasciclin and AGP domains (one or two), and the presence of a glycosylphosphatidylinositol (GPI) anchor, a total of 21 FLAs have been identified and divided into four subgroups from A to D (Johnson *et al.*, 2003).

Although FLA proteins are widely spread in the plant kingdom, little of their biological function is fully clarified to date. In *Arabidopsis*, FLA4 is involved in salt tolerance, normal root expansion, and the structure of seed coat mucilage (Shi *et al.*, 2003; Harpaz-Saad *et al.*, 2011). In the *Atfla11*/*12* double T-DNA insertion mutant, stem tensile strength and stiffness are decreased due to alteration of the cell wall matrix (MacMillan *et al.*, 2010). In cotton, *GhFLA1* plays an important role in fibre initiation and elongation by affecting AGP composition and the integrity of the primary cell wall matrix (Huang *et al.*, 2013). In trees, some FLA proteins are associated with wood formation and are expressed preferentially in differentiating xylem, although their precise functions are yet to be further elucidated (No and Loopstra, 2000; Whetten *et al.*, 2001; Lorenz and Dean, 2002; Qiu *et al.*, 2008; Dharmawardhana *et al.*, 2010).

Transcriptomic analyses have been performed to study the molecular mechanism of wood formation in *Populus* (Déjardin *et al.*, 2004; Sterky *et al.*, 2004; Andersson-Gunnerås *et al.*, 2006; Dharmawardhana *et al.*, 2010). A group of *FLA* genes (*PopFLA1–10*, also named *PtFLA12D*, *-E*, *-F*, *-G*, *-J*, -*K*, *-P*, *-Q*, -*S*, and *-V*) are highly upregulated in tension wood, and specifically accumulate in the secondary xylem tissues of poplar stem (Lafarguette *et al.*, 2004; Andersson-Gunnerås *et al.*, 2006). The expression pattern of these poplar *FLA*s implies their important but still unknown functions in wood formation. We have been studying the functional analysis of these *FLA*s in regular and tension wood formation in aspen. In this study, PtFLA6 expression and distribution were investigated, and transgenic poplar plants expressing the antisense cDNA of *PtFLA6* driven by its own promoter were generated. We found that *PtFLA6* was expressed predominantly in the xylem fibre cells in poplar. Antisense expression of *PtFLA6* decreased the transcripts of *PtFLA6* and its homologous genes, and reduced the amount of PtFLA6 and AGPs in transgenic plants, leading to altered cell wall composition.

## Materials and methods

### Plant materials and growth conditions

*Populus trichocarpa* genotype Nisqually-1 and a commercial clone Shanxin yang (*Populus davidiana×Populus bolleana*) were used in this study. Generally, *in vitro*-grown plants were subcultured monthly by aseptically transferring shoot apices to fresh MS medium supplemented with 0.1mg l^–1^ of 1-naphthaleneacetic acid (Murashige and Skoog, 1962). Plantlets were also transferred into individual pots and grown in a greenhouse under a 12h light/12h dark photoperiod, comprising natural daylight supplemented with lamps (Philips, The Netherlands) to give a minimum quantum ﬂux density of ~200 µmol m^–2^ s^–1^. The temperature was kept at 21–25 °C in daytime and 15–18 °C at night. All plants were well watered according to the evaporation demands during different growth stages, and fertilized biweekly with water-soluble fertilizers (Plant-Soul, China).

### Quantitative real-time RT-PCR

For the expression pattern analysis of *PtFLA6* in poplar, total RNA was extracted from different organs and tissues of 3-month-old plants, comprising apical buds, mature leaves, leaf petioles, roots, xylem and phloem tissues of upper, middle, and basal stems, with the RNAiso Reagent (Takara, Japan). After treatment with DNase I (Promega, USA), 2 μg of total RNA was subjected to reverse transcription using a RevertAid First Strand cDNA Synthesis kit (Thermo Scientific, USA) at 42 °C for 1h. The resultant cDNA was then used for quantitative real-time RT-PCR (qRT-PCR) with gene-specific primers (Supplementary Table S1 at *JXB* online). qRT-PCR was performed using an AceQ qPCR SYBR Green Master Mix (Vazyme Biotech, China) and a CFX Connect Real-Time System (Bio-Rad, USA). The relative expression of each target gene was normalized using the housekeeping gene *PtEF1β* (Brunner *et al.*, 2004). The log_2_ fold change in value was calculated based on 2^–ΔCt^ method.

For the expression analysis of *PtFLA*s and poplar secondary cell wall biosynthesis-related genes, xylem tissues of middle stems from 3-month-old wild type (WT) and transgenic plants grown in a greenhouse were used for RNA extraction. The gene-specific primers used for qRT-PCR analyses are shown in Supplementary Table S1. The elongation factor gene *PtEF1β* was used as an internal control in all experiments unless specifically indicated. The expression value of these genes in the WT was set to 1.

### Antibody production and western blotting

For antibody production, PtFLA6 protein without the N-terminal signal peptide (24 aa) was fused with the glutathione *S*-transferase (GST) tag in the pGEX-4T-1 expression vector. The fusion protein (about 53kDa) was purified from *Escherichia coli* culture and used to raise a polyclonal antibody in rabbit (ABclonal, USA). Crude antisera were purified using a protein A–Sepharose Cl-4B column. Anti-plant actin monoclonal antibodies and secondary antibodies were purchased from ABclonal.

For western blot assays, plant proteins were extracted with a buffer consisting of 50mM Tris/HCl (pH 8.0), 10mM EDTA, and 1% (w/v) Triton X-100, and separated by 10 % SDS-PAGE. After electrotransfer of the proteins onto polyvinylidene difluoride membranes, the membranes were blocked with Tris-buffered saline (TBS) (10mM Tris/HCl, pH 7.5, 0.1% NaCl) supplemented with 5% non-fat dried milk for 1h. The membranes were incubated with anti-PtFLA6 antibodies (diluted at 1:1000) in TBST buffer (10mM Tris/HCl, pH 7.5, 0.1% NaCl, 0.05% Tween 20) containing 1% non-fat dried milk for 1h at room temperature or overnight at 4 °C. Afterwards, the membranes were rinsed three times with TBST buffer and incubated with the secondary antibodies (peroxidase-labelled anti-rabbit antibodies) at a dilution of 1:5000 for 1h. After washed three times with TBST buffer (5min each), the membranes were incubated in LumiGLO for chemiluminescence detection (KPL, USA) and then imaged with a Tanon 5500 electrophoresis system (Tanon, China).

For western blot analysis of PtFLA6, total proteins were extracted from phloem and xylem tissues of the stems of 3-month-old greenhouse-grown poplar plants. The middle parts of stems were used because of the high expression of *PtFLA6* and the easy grinding of the materials into a fine powder in liquid nitrogen compared with the basal parts of the stems. For comparison of PtFLA6 between WT and transgenic plants, total proteins from peeled stems of the middle parts of WT and transgenic plants were used.

### Immunolocalization of PtFLA6

For immunohistochemical analysis, upper, middle, and basal parts of poplar stems were fixed overnight in 0.1M PBS (pH 7.5) containing 4% paraformaldehyde, and embedded in paraffin. The slides were spread with polylysine before the sections were fixed. After deparaffinization and dehydration, the sections were washed twice with PBS buffer. The samples were blocked with 5% bovine serum albumin (BSA) in culture medium for 1h at room temperature. Subsequently, they were incubated with anti-PtFLA6 antibodies (diluted at 1:50 with 0.1M PBS containing 0.1% BSA) at room temperature for 1h. In the negative control, pre-immune serum was substituted for PtFLA6 antibody. After rinsing three times in PBS (5min each), the samples were incubated with alkaline phosphatase-conjugated secondary antibody (diluted at 1:100 in the same buffer) at room temperature for 1h. After a final rinse in PBS, the samples were developed for approximately 1h by adding 150–200 µl of Western Blue stabilized substrate for alkaline phosphatase (Promega, USA). When the blue colour appeared on the sections, they were rinsed with water and mounted with a cover glass for photographing. Images were captured under a bright field using an ECLIPSE 80i microscope (Nikon, Japan).

For immunogold-labelling transmission electron microscope (TEM) analysis, sections on grids were blocked for 1h with blocking solution, which consisted of 50mM TBS (pH 7.5), 3% BSA and 0.01% Tween 20. The sections were incubated with rabbit anti-PtFLA6 antibodies (diluted 1:50). The grids were washed with TBST buffer (pH 7.5) and incubated for 1h with goat anti-rabbit gold conjugate (10nm; Sigma, USA) (diluted 1:50). Finally, the grids were washed with TBST and water before staining with 2% aqueous uranyl acetate. Control sections were obtained by omitting the primary antibody.

### Subcellular localization of PtFLA6

To determine the subcellular localization of PtFLA6 protein, the encoding region without the stop codon of PtFLA6 was fused in frame to the N terminus of yellow fluorescent protein (YFP) in the pA7-YFP vector. The final plasmid, pA7-YFP or pA7-PtFLA6-YFP, was transfected into Shanxin yang mesophyll protoplasts, essentially as described previously (Sheen, 2001; Yoo *et al.*, 2007). After transfection with the relative plasmid, protoplasts were incubated at 23 °C for 16h and analysed for PtFLA6–YFP expression using a confocal microscope at 514nm (Zeiss LSM 510; META, Germany). YFP (514nm) was used as a positive control. Data were processed using Photoshop software (Adobe, USA).

### Plasmid vector and plant transformation

To construct the antisense expression vector, the full-length coding sequence of *PtFLA6* and the 1.4kb promoter region of *PtFLA6* were cloned from Nisqually-1 using gene (*PtFLA6-OF* and *PtFLA6-OR*) and promoter (*PtFLA6-PF* and *PtFLA6-PR*) primers, respectively (Supplementary Table S1). After sequence confirmation, the coding sequence of *PtFLA6* was inserted into the modified pCAMBIA-2301 vector in an antisense orientation (Huang *et al.*, 2009). The 35S promoter in anti-*PtFLA6* was replaced with the *PtFLA6* promoter. The resultant construct *PtFLA6Pro*–anti-*PtFLA6* was introduced into Shanxin yang by *Agrobacterium*-mediated transformation as described previously (Wang *et al.*, 2011). Regenerated shoots were analysed with β-glucuronidase staining, PCR and RT-PCR. For PCR and RT-PCR analyses, the forward promoter primer and *PtFLA6* primer (as reverse primer) or gene-specific primers were used (Supplementary Table S1). Transgenic plants were propagated, transferred to soil, and grown in a greenhouse for further study.

### Microscopic experiments

For morphological observations, the middle stems from 3-month-old WT and transgenic plants grown in a greenhouse were ﬁxed with 2% formaldehyde as described previously (Wang *et al.*, 2013). For TEM observation, stems from WT and transgenic plants were fixed in glutaraldehyde solution and embedded in EPON 812 resin (Shell, USA). Sections (70–80nm) were cut and harvested on grids, stained with uranyl acetate and lead citrate, and observed with an H-7650 electron microscope (Hitachi, Japan).

### AGP content assays

Total proteins were extracted from the stems of WT and transgenic plants as described previously (Schultz *et al.*, 2000). Briefly, 10g (fresh weight) of xylem tissue of poplar stem was ground into a fine powder in liquid nitrogen, and 10ml of extraction buffer (50mM Tris/HCl, pH 8.0, 10mM EDTA, 0.1% β-mercaptoethanol, and 1%, w/v, Triton X-100) was added. After incubated at 4 °C for 16h, the samples were centrifuged for 10min at 14 000*g*. The supernatant was collected and precipitated with 5 vols of ethanol at 4 °C for 12h. The pellet was resuspended in 5ml of 50mM Tris/HCl (pH 8.0). Proteins were quantified with a BCA Protein Assay kit (Thermo, USA).

The content of AGPs in the proteins extracted from 3-month-old poplar stem was quantified by rocket electrophoresis as reported previously, with minor modifications (Zhu *et al.*, 1993). A total of 20 μg of protein extract was analysed in 1% (w/v) agarose gel (1.0mm thick) containing 0.025M Tris, 0.2M glycine, and 10 μg ml^–1^ of β-glucosyl Yariv reagent (Biosupplies, Australia). Electrophoresis was performed in the same buffer for 6h at 5V cm^–1^. After completion of electrophoresis, the gel was washed with 1% (w/v) NaCl, rinsed with distilled water, and dried in a warm air stream. Gum arabic (Sigma, USA) was used as a standard.

### Wood-bending tests

Stems of 3-month-old WT and transgenic plants grown in a greenhouse were used for mechanical tests as described previously (Kasal *et al.*, 2007; Yu *et al.*, 2014). Briefly, the basal segments of stems were oven dried at 50 °C and used for flexural three-point bending stiffness and strength tests. The tests were performed using a mechanical testing machine (HY-0580, China) equipped with a data acquisition system at room temperature (~25 °C).

### Cellulose and lignin quantification

The middle stems of 3-month-old WT and transgenic plants were cut into small pieces and ground into a fine powder in liquid nitrogen. Crystalline cellulose and lignin content was determined as described previously (Foster *et al*., 2010*a*, *b*).

### Statistics

All data in this work were obtained from at least three independent experiments with three replicates each. Data were analysed using one-way analysis of variance followed by Duncan’s multiple range test (*P*<0.01). GenBank accession numbers and gene models in this study were listed in Supplementary Table S2 at *JXB* online.

## Results

### *PtFLA6* and its homologous genes are xylem specific in poplar

**Fig. 1.**
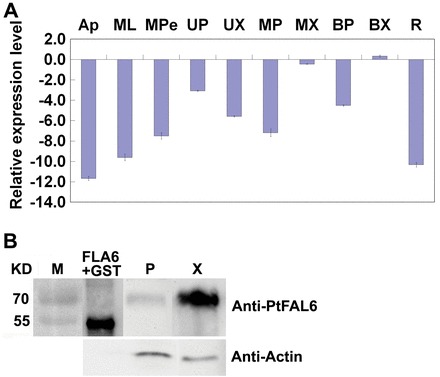
Expression analysis of *PtFLA6*. (A) qRT-PCR analysis of *PtFLA6* in poplar. The *y*-axis represents the log_2_ fold change of *PtFLA6* in various tissues of poplar compared with *PtEF1β*. AP, apex; ML, mature leaf; MPe, mature leaf petiole; UP, upper stem phloem; UX, upper stem xylem; MP, middle stem phloem; MX, middle stem xylem; BP, basal stem phloem; BX, basal stem xylem; R, root. Experiment was repeated three times using different batches of plants. (B) Western blot analysis of PtFLA6 in poplar stem. Total proteins were extracted from the phloem and xylem of middle stems of poplar. About 20 μg of protein was electrophoresed by 10% SDS-PAGE and hybridized with anti-PtFLA6 antibodies. M, protein molecular weight marker; FLA6+GST, fusion protein; P, protein isolated from the phloem of middle stems; X, protein isolated from the xylem of middle stems. (This figure is available in colour at *JXB* online.)

Based on the nucleotide sequences of *PopFLA1–10* (Lafarguette *et al.*, 2004), the encoding sequences of *PtFLA*s were extracted from the Joint Genome Initiative poplar database (*Populus trichocarpa* genome portal v.1.1, http://genome.jgi-psf.org/Poptr1_1/Poptr1_1.home.html) and named *PtFLA1–10*, respectively. All *PtFLA*s shared very high similarity, and *PtFLA2* and *PtFLA3* had exactly the same nucleotide sequence (Supplementary Fig. S1 at *JXB* online). To elucidate the possible roles of these PtFLAs in poplar, *PtFLA6*, which displayed high identity with *AtFLA11* and *AtFLA12* (Supplementary Fig. S2 at *JXB* online), was chosen for further study, and its expression pattern in Shanxin yang was investigated with qRT-PCR. We observed that *PtFLA6* was expressed specifically in the stems rather than in other tissues such as shoot apexes, leaves and roots, and was predominantly expressed in the xylem tissues of middle and basal stems, where secondary cell wall formation was active (Fig. 1A). A similar expression pattern was also observed with the other nine *PtFLA*s (Supplementary Fig. S3 at *JXB* online).

To see whether the specific and high expression of *PtFLA6* in the xylem tissues would consequently lead to a high accumulation of PtFLA6 protein, the N-terminal signal peptide was removed and PtFLA6 antibody was produced. Western blot analysis showed that a large amount of PtFLA6 protein accumulated in the xylem but only a trace amount of PtFLA6 protein was detected in the phloem tissues of mature stems (Fig. 1B).

**Fig. 2.**
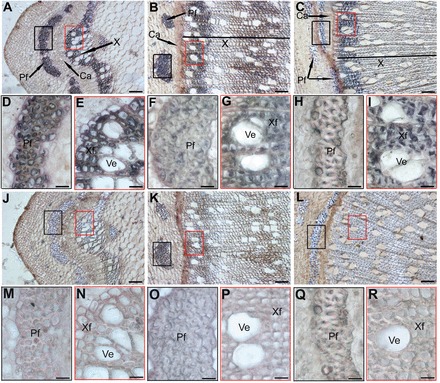
Immunolocalization of PtFLA6 in the stems of poplar. (A–I) Cross-sections of stems at the upper, middle, and basal positions of greenhouse-grown plants were hybridized with anti-PtFLA6 antibodies, respectively. (D) and (E), (F) and (G), and (H) and (I) show high magnification of the framed areas in (A), (B), and (C), respectively. (J–R) Negative controls of (A), (B), and (C) hybridized with pre-immune IgG. (M) and (N), (O) and (P), and (Q) and (R) show high magnification of the framed areas in (J), (K), and (L), respectively. Pf, phloem fibre cell; Ca, cambia; X, xylem; Ve, vessel cell; Xf, xylem fibre cell. Blue staining indicates PtFLA6 signals. Bars, 100 μm (A–C, J–L); 25 μm, (D–I, M–R). (This figure is available in colour at *JXB* online.)

In mature stem of poplar, the xylem is composed of differentiating, expanding, secondary-wall formation and mature xylem (Schrader *et al.*, 2003; Israelsson *et al.*, 2005). Primary phloem and xylem are present in the upper stem of poplar, and secondary phloem and xylem are produced in the mature stem (Dharmawardhana *et al.*, 2010). In order to understand the precise tissue distribution of PtFLA6, immunolocalization of PtFLA6 in the stems was performed (Fig. 2). Stem sections from the upper, middle, and basal parts of poplar were hybridized with antibodies against PtFLA6 or pre-immune serum (negative control). Strong blue signals for PtFLA6 were detected in the primary phloem fibres and primary xylem of upper stems, and in the secondary xylem of middle and basal stems, where the secondary wall was forming (Fig. 2A–C), but no signal was detected in the control samples (Fig. 2J–L). Further analysis revealed that PtFLA6 was present mainly in the primary phloem fibre cells of upper stems, marginally present in the secondary phloem fibres of middle stems, and absent in those of basal stems (Fig. 2D, F, H). Most of all, strong PtFLA6 signals were detected in the primary xylem fibres of upper stems and in the secondary-wall formation zone of xylem (stems at middle and basal positions) (Fig. 2E, G, I). Again, no signal was detected in the negative controls (Fig. 2M–R). These results implied that PtFLAs may play important roles during the secondary wood formation of poplar.

### PtFLA6 protein is accumulated in fibre cells

**Fig. 3.**
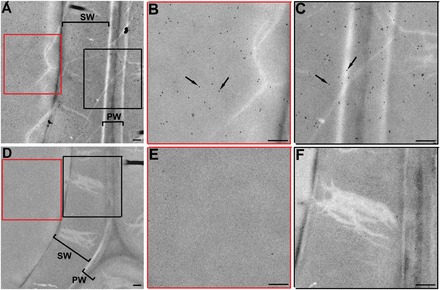
Immunocytolocalization of PtFLA6 in the xylem fibre cells of poplar stem. Transverse ultrathin sections of the xylem cells from 3-month-old poplar stems were used for immunogold-labelling TEM analysis. (A) A PtFLA6 signal was observed in both the cytoplasm and cell wall of xylem fibre cells. (B, C) High magnification of the framed areas in (A). (D) Negative control of (A). No signal was detected. (E, F) High magnification of the framed areas in (D). Black dots indicated by arrows in (B) and (C) indicate the immunogold signallings. PW, primary wall; SW, secondary wall. Bars, 250nm. (This figure is available in colour at *JXB* online.)

Mature xylem tissues are composed of a large number of mature fibre cells, and their walls consist of biomacromolecules such as lignin, cellulose, pectins, and proteins. To determine whether PtFLA6 also accumulated in the cell wall of xylem fibres, immunogold-labelling TEM analysis was performed. Immunogold signals for PtFLA6 were detected in both the cytoplasm and cell wall (primary and secondary wall) of xylem fibre cells (Fig. 3A–C), in contrast to the negative controls in which no signal was observed (Fig. 3D–F).

**Fig. 4.**
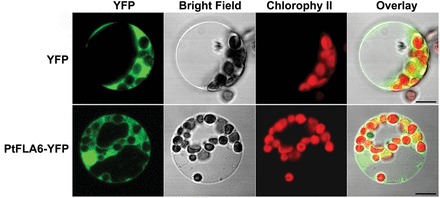
Subcellular localization of PtFLA6 protein. PtFLA6 or YFP alone were transiently expressed in the protoplasts of Shanxin yang, incubated in dark for 12h and observed with a confocal microscope. Bar, 5 μm. (This figure is available in colour at *JXB* online.)

It has been reported that most AGPs are localized in the plant cell wall and plasma membrane (reviewed by Ellis *et al.*, 2010). Since subcellular localization is crucial for the proper function of most proteins, we examined the subcellular localization of PtFLA6 in poplar mesophyll protoplasts. YFP-tagged PtFLA6 was transiently expressed in mesophyll protoplasts isolated from poplar leaves. Confocal imaging showed that the fluorescence signals of the PtFLA6–YFP fusion protein coincided precisely with the ubiquitous distribution of free YFP (Fig. 4).

### Antisense expression of *PtFLA6* affects stem biomechanics in transgenic poplar

**Fig. 5.**
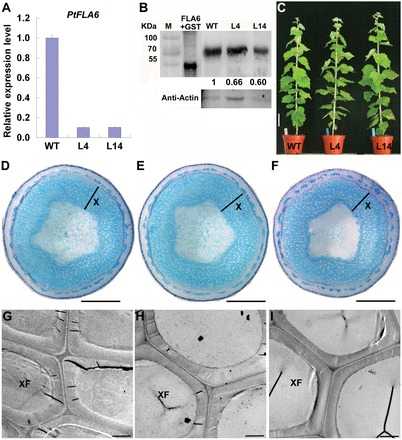
Molecular, phenotypic, and histochemical analysis of *PtFLA6* antisense transgenic plants. (A) qRT-PCR analysis of *PtFLA6* in the xylem of WT and *PtFLA6* antisense transgenic plants (lines L4 and L14). Total RNA was isolated from the xylem tissues of 3-month-old plants grown in a greenhouse. The experiment was performed three times using different batches of plants. Error bars represent the standard deviation (SD) of three technical replicates using pooled samples of three independent plants. The expression level of *PtFLA6* in the xylem of the WT was set to 1. (B) Western blot analysis. Total proteins were extracted from the xylem tissue in WT and transgenic plants. About 20 μg of protein was separated by 10% SDS-PAGE, and hybridized with anti-PtFLA6 and anti-actin antibodies, respectively. Expression of PtFLA6 in the xylem of the WT was set to 1. M, protein molecular weight marker; FLA6+GST, fusion protein for antibody production; WT, protein isolated from the xylem of WT plants; L4 and L14, protein isolated from the xylem of transgenic lines L4 and L14. (C) Phenotypes of 3-month-old WT and transgenic plants (lines L4 and L14) grown in a greenhouse. Bar, 10cm. (D–F) Cellular morphology of the stems from the WT (D) and transgenic lines L4 (E) and L14 (F). Transverse sections of the middle stems were stained with 0.05% toluidine blue. X, xylem. Bars, 1mm. (G–I) Transmission electron micrographs of xylary ﬁbres in the stem of the WT (G) and transgenic lines L4 (H) and L14 (I). XF, xylem fibre cell. Bars, 5 μm. (This figure is available in colour at *JXB* online.)

The xylem-specific expression of the *PtFLA6* gene and the location of PtFLA6 protein in xylem fibre cells, suggested its possible participation in wood formation in poplar. Therefore, the antisense cDNA of *PtFLA6* was expressed in poplar via *Agrobacterium tumefaciens-*mediated transformation under the control of its own promoter (Supplementary Fig. S4A at *JXB* online). Twenty-two independent kanamycin-resistant lines were regenerated, and seven lines were selected for β-glucuronidase staining and PCR analysis (Supplementary Fig. S4B, C). The transcripts of *PtFLA6* were suppressed to different levels in the stems of these transgenic lines (Supplementary Fig. S4D). Among them, lines L4 and L14, which showed the most significant suppression of *PtFLA6* expression, were chosen for this study. After growth for 3 months in a greenhouse, the xylem tissues of WT and transgenic plants were taken for qRT-PCR and Western blot analyses. As shown in Fig. 5A, a significant decrease in *PtFLA6* transcripts (Fig. 5A) and PtFLA6 protein (Fig. 5B) was observed in the xylem tissues of both transgenic lines. Antisense expression of *PtFLA6* did not cause any phenotypic changes since both WT and transgenic plants grew normally in the greenhouse (Fig. 5C). No significant difference was seen between the WT and transgenic plants, such as stem height and diameter (Supplementary Fig. S5 at *JXB* online), xylem development (Fig. 5D–F), and xylem fibre cell wall thickness (Fig. 5G–I), as confirmed with toluidine blue staining and TEM analyses.

**Fig. 6.**
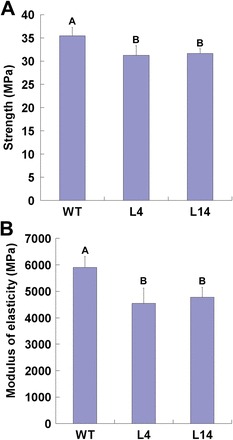
Biomechanical analysis of WT and transgenic plants (lines L4 and L14). (A) Flexural strength test. (B) Flexural stiffness test. Basal stems of WT and transgenic plants were used. Values are means±SD of 10 independent plants of WT and each transgenic line. Values labelled with different letters are significantly different (*P*<0.01). (This figure is available in colour at *JXB* online.)

Previously, reduced stem tensile strength and stiffness were observed in the *Atfla11*/*12* double mutant (MacMillan *et al.*, 2010). To see whether suppressed expression of *PtFLA6* would also affect the stem biomechanical properties in trees, poplar plants grown in a greenhouse with obvious secondary xylem tissues were subjected to three-point stem biomechanical tests. A 10–12% reduction in stem flexural strength was observed in transgenic plants (Fig. 6A). In addition, the stem flexural stiffness (modulus of elasticity) of the tested transgenic plants was reduced by 19–23% compared with that of the WT plants (Fig. 6B). Therefore, downregulated expression of *PtFLA6* altered the stem physical properties in transgenic poplar plants.

### Expression of PtFLA6 homologues is also downregulated in transgenic plants

**Fig. 7.**
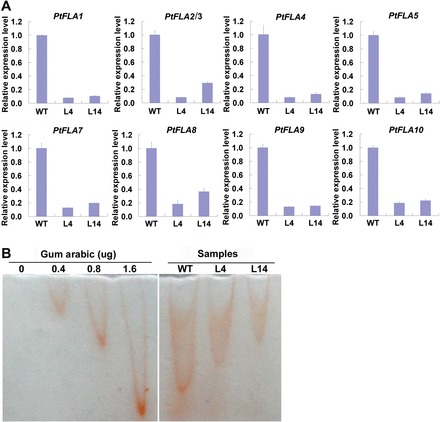
Gene expression and AGP composition analyses of WT and transgenic plants (lines L4 and L14). (A) Expression of the other nine *PtFLA*s in the xylem tissues of WT and transgenic plants. Total RNA was isolated from the stems of 3-month-old plants grown in a greenhouse. The experiment was performed three times using different batches of plants. Error bars represent the SD of three technical replicates using pooled samples of three independent plants. The expression values of the WT were set to 1. (B) AGP content analysis. Rocket Yariv agarose gel electrophoresis was performed. Gum arabic or plant proteins, extracted from the xylem tissues of the WT and transgenic plants, were run on an agarose gel containing 10 µg ml^–1^ of β-glucosyl Yariv reagent. Concentrations of 0, 0.4, 0.8, and 1.6 µg of gum arabic were used as standard. The peak area represents the content of AGPs. (This figure is available in colour at *JXB* online.)

Since *PtFLA6* shares very high homology and a similar expression pattern with the other nine *PtFLA*s (Fig. 1A and Supplementary Figs S1 and S3), and the native promoter of *PtFLA6* was used to drive the antisense expression of *PtFLA6* (Supplementary Fig. S4A), we further evaluated the expression of the other nine *PtFLA*s in these transgenic lines. qRT-PCR analysis was performed with gene-specific primers (Supplementary Table S1). As expected, expression of these homologous *PtFLA*s was also significantly downregulated in the xylem tissues of transgenic plants (Fig. 7A).

### Transgenic plants accumulate less APGs

In the light of reduced expression of *PtFLA6* and its homologous genes in transgenic plants, we hypothesized that the production of APGs might also be affected. Therefore, we compared the WT and transgenic plants for their AGP content in the xylem tissues. Rocket β-glucosyl Yariv agarose gel electrophoresis was carried out. The results revealed that protein samples from the xylem tissues of transgenic plants formed a smaller peak area than the WT samples, which meant that AGPs and other products in the stems of transgenic plants were reduced (Fig. 7B).

### Antisense expression of PtFLA6 alters secondary cell wall composition

**Fig. 8.**
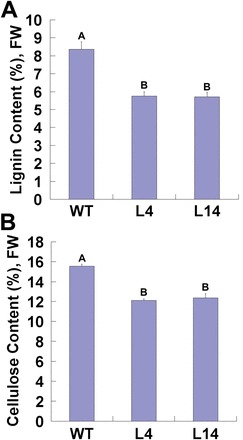
Comparison of lignin and cellulose content in the xylem tissues of the WT and transgenic plants (lines L4 and L14). (A) Lignin content. (B) Cellulose content. Three individual plants were sampled for each transgenic line. Error bars show SD (*n*=3). Values labelled with different letters are significantly different (*P*<0.01). FW, fresh weight. (This figure is available in colour at *JXB* online.)

**Fig. 9.**
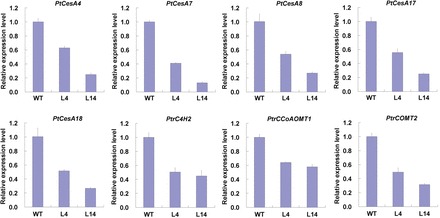
qRT-PCR analysis of secondary cell wall biosynthesis genes in the WT and transgenic lines L4 and L14. Total RNA was isolated from the stem xylem tissues of 3-month-old plants grown in a greenhouse. Expression of both cellulose biosynthesis (*PtCesA4*, *PtCesA7*, *PtCesA8*, *PtCesA17*, and *PtCesA18*) and lignin biosynthesis (*PtrC4H2*, *PtrCCoAOMT1*, and *PtrCOMT2*) genes was analysed. The experiment was performed three times using different batches of plants. Error bars represent the SD of three technical replicates using pooled samples of three independent plants. The expression level of genes in the WT was set to 1. (This figure is available in colour at *JXB* online.)

Lignin and cellulose are two major components in the secondary xylem cell walls. To understand whether reduced stem flexural strength and flexural modulus were a consequence of altered biosynthesis of the cell wall matrix in the stems of transgenic plants, content of lignin and cellulose was determined in the WT and transgenic lines L4 and L14. As shown in Fig. 8, the content of both lignin (Fig. 8A) and crystalline cellulose (Fig. 8B) in the xylem tissues of transgenic plants was dramatically lower than that in the xylem tissues of WT plants. In *Populus*, lignin and cellulose biosynthesis-related genes have been well characterized (Suzuki *et al.*, 2006; Shi *et al.*, 2010). Therefore, we examined the expression of some xylem-specific genes involved in lignin and cellulose biosynthesis by qRT-PCR. Five cellulose biosynthesis genes (*PtCesA4*, *PtCesA7*, *PtCesA8*, *PtCesA17*, and *PtCesA18*) and three lignin biosynthesis genes (*PtrC4H2*, *PtrCCoAOMT1*, and *PtrCOMT2*) were analysed. We found that all these genes were significantly downregulated in the transgenic lines L4 and L14 compared with the WT controls (Fig. 9). These results suggested that suppression of *PtFLA6* and its homologues also modulated the biosynthesis of the secondary cell wall during wood formation in these transgenic plants.

## Discussion

In higher plants, FLAs are highly and specifically expressed in stems undergoing secondary cell wall deposition in *Arabidopsis* (MacMillan *et al.*, 2010), poplar (Lafarguette *et al.*, 2004), and zinnia (Dahiya *et al.*, 2006). However, the biological functions of FLAs in trees are largely unclear. The present study reports functional analyses of *PtFLA6* and its homologues in poplar.

The cell wall matrix of secondary xylem fibres is composed of cellulose, hemicellulose, lignin, and wall proteins (Cosgrove, 1997). Among these, cell wall proteins play important roles in the maintenance of physical and biological functions of the plant cell wall network, and AGPs are the major components of cell wall proteins. PtFLA proteins, a subclass of AGPs, accumulated abundantly in xylem tissues (Figs 1B and 2A–I). Therefore, PtFLAs may have important roles in wood and cell wall formation of poplar plants. Our observations that *PtFLA6* and its homologous genes were expressed specifically in the xylem tissues of mature stems undergoing secondary wall formation (Fig. 1A and Supplementary Fig. S3) and that PtFLA proteins accumulated abundantly in xylem fibre cell walls support this hypothesis (Figs 1B, 2A–I, and 3A–C). It is also noteworthy that the molecular weight of mature PtFLA6 detected in poplar stem was larger than that of its native protein, which is only about 26kDa (Fig. 1B). This could be the result of glycosylation during maturation of the PtFLA6 protein since FLAs are a subclass of AGPs which are highly and complexly glycosylated in their protein backbones (Johnson *et al.*, 2003). Indeed, five *N*-glycosylation sites and 11 *O*-glycosylation sites were predicted using the network prediction of glycosylation sites in PtFLA6 (Supplementary Fig. S6 at *JXB* online).

A signal peptide (SP) at the N terminus and a complete C-terminal signal for the GPI anchor have been predicted in PtFLA6 and its homologues in poplar and *Arabidopsis* (Lafarguette *et al.*, 2004; MacMillan *et al.*, 2010). AGP proteins are guided into the endoplasmic reticulum by the SP, where the GPI anchor is synthesized, and then added on to the C terminus of the mature protein by a transamidation reaction. Extensive lipid remodelling of the GPI anchor is completed within the Golgi. The matured AGPs are then transported to the plasma membrane by vesicular transport (Ellis *et al.*, 2010). It has been confirmed that the SP and GPI-anchored signal of the GhFLA1 protein can localize green fluorescent protein (GFP) fluorescence between the cell wall and plasma membrane (Huang *et al.*, 2013), but GFP was localized in the cytoplasm when GFP was fused to the C terminus of GhFLA1 with a truncated GPI anchor. Therefore, the GPI-anchoring signal is important for the proper localization of AGPs.

In this study, YFP fused to the C terminus of PtFLA6 could have blocked the addition of the GPI anchor, leading to a ubiquitous distribution of PtFLA6–YFP fusion protein (Fig. 4). Indeed, when cyan fluorescent protein was fused to the N terminus of PtFLA6, similar results were also observed (data not shown). One possible reason is that the SP recognition site was affected and blocked the transfer of PtFLA6 to the endoplasmic reticulum for addition of the GPI anchor. Therefore, the fluorescent protein fused to either the N or C terminus of PtFLA6 could have influenced its native localization. Another possibility is that the proper localization of PtFLA6 may depend on the specific developmental stage and specific tissue. Our immunogold-labelling TEM analysis confirmed that PtFLA6 is a cell wall protein (Fig. 3). The subcellular location of PtFLA6 in our study further verified the importance of the SP and GPI in PtFLA6-like proteins, as reported previously (Huang *et al.*, 2013).

In addition to the similar expression patterns of *PtFLA*s, their DNA sequences are also highly similar (Supplementary Fig. S1). Therefore, they may have redundant functions in poplar. To answer this question, the full-length coding sequence of *PtFLA6* was used to construct an antisense vector. As expected, antisense expression of *PtFLA6* successfully reduced the transcription of all *PtFLA*s including itself (*PtFLA1*, *PtFLA2/3*, *PtFLA4*, *PtFLA5*, *PtFLA7*, *PtFLA8*, *PtFLA9*, and *PtFLA10*) (Figs 5A and 7A), and as a consequence decreased the production of PtFLA6 and AGPs in transgenic plants (Figs 5B and 7B). The downregulated production of PtFLAs did not affect the normal growth and xylem differentiation in transgenic plants (Fig. 5C–I and Supplementary Fig. S5). Therefore, PtFLAs may be not involved in stem division. This is also supported by our observation that *PtFLA*s were expressed predominantly in the xylem tissues undergoing secondary-wall formation but not in the cambia of mature poplar stems (Fig. 2A–C). However, stem physical properties were altered in transgenic plants, possibly due to the reduced amount of PtFLAs and AGPs. Indeed, stem biomechanics in transgenic plants were obviously changed with weaker stem strength and stiffness (Fig. 6). However, the changes were slight. Possible explanations are that poplar plants grown in a greenhouse are weaker than those grown in field and that the stems used for three-point tests were too young. We transferred the plants to the field for the measurement of mechanical properties in mature stems in our further studies. In addition, there exist hundreds of FLA-like expressed sequence tags in the *Populus* genome, and of these, over 200 genes belong to the subgroup of *PtFLA*s (Andersson-Gunnerås *et al.*, 2006). Thus, the remnants of these PtFLAs may still function effectively in transgenic plants. This inference is also supported by our western blot results, which showed that PtFLA6-like proteins were only reduced by about 30–40% in transgenic plants (Fig. 5B). Furthermore, AGPs are a superfamily of cell wall glycoproteins/proteoglycans, which are also present in xylem fibre cell walls (Lafarguette *et al.*, 2004; Ellis *et al.*, 2010). Therefore, other AGPs may still function sufficiently to maintain the normal mechanical properties of transgenic plants.

Lignin and cellulose are two major cell wall components of secondary xylem cells. Reduction of PtFLAs in xylem cells also affected cell wall matrix deposition in transgenic plants, although the secondary cell wall thickness of transgenic plants was not changed (Fig. 5G–I). The content of lignin and cellulose decreased dramatically in the xylem of the transgenic lines L4 and L14 (Fig. 8), and some of xylem-specific genes associated with cellulose biosynthesis (*PtCesA4*, *PtCesA7*, *PtCesA8*, *PtCesA17*, and *PtCesA18*) (Suzuki *et al.*, 2006) and lignin biosynthesis (*PtrC4H2*, *PtrCCoAOMT1*, and *PtrCOMT2* (Shi *et al.*, 2010) were downregulated in transgenic plants (Fig. 9). Therefore, suppressed expression of PtFLAs may have restrained the transcription of secondary-wall biosynthesis genes and as a result, altered the composition of cell wall components during the secondary growth, leading to decreased stem flexural strength and stiffness in transgenic plants. Since FLAs contain one or more cell-adhesion domains (fasciclin domain) that work(s) as adhesion molecules between the macromolecules of the xylem secondary cell wall, by attaching to the plasma membrane, PtFLAs could play a signalling role to regulate the cell wall biosynthesis genes via interaction with cell wall-associated kinases.

Unlike the *Atfla11/fla12* double mutant, in which tensile strength and elasticity were reduced (MacMillan *et al.*, 2010), the altered biomechanics in transgenic poplar plants were due to the reduced flexural strength and stiffness (Fig. 6), which were increased in *Atfla11*, *Atfla12*, and *Atfla11/fla12* mutants. Therefore, the structure and composition of secondary wood in trees may have some specific features, although *Arabidopsis* also undergoes a certain degree of secondary formation (Ye *et al.*, 2002). We also tried to measure the stem tensile strength and elasticity but failed because poplar stems are very easy to break at the holding sites.

Lignin has been shown to be very important in providing flexural strength (MacKay *et al.*, 1997; MacMillan *et al.*, 2010). Acid-insoluble lignin content in the stems of *Atfla11*, *Atfla12*, and *Atfla11/fla12* mutants was higher than in the WT (MacMillan *et al.*, 2010). Thus, it is conceivable that transgenic poplar plants with reduced flexural strength and stiffness produced less lignin (Figs 6A and 8A). Cellulose content has been thought to be a major factor affecting tensile strength (Li *et al.*, 2003; MacMillan *et al.*, 2010). In this study, we found that transgenic plants also accumulated less cellulose in their xylem tissues (Fig. 8B). Therefore, a cellulose content decrease also contributed to the reduction in flexural strength and elasticity in transgenic plants (Fig. 6B). Taken together, antisense expression of *PtFLA6* and its homologous genes altered cell wall composition, leading to reduced stem flexural strength and stiffness in transgenic poplar. Our findings reported here will aid in future trials to engineer this quality trait by manipulating the biosynthesis of AGPs in poplar, and possibly also in other woody plants.

## Supplementary material

Supplementary data are available at *JXB* online.

Supplementary Table S1. Primers used in this study.

Supplementary Table S2. GenBank accession numbers or gene models used in this study.

Supplementary Fig. S1. DNA sequence alignment of *PtFLA1–10* from *Populus trichocarpa*.

Supplementary Fig. S2. Phylogenetic analysis of DNA sequences of *PtFLA*s and *AtFLAs*.

Supplementary Fig. S3. qRT-PCR analysis of the other nine *PtFLA*s.

Supplementary Fig. S4. Molecular confirmation of transgenic plants.

Supplementary Fig. S5. Phenotypic analysis.

Supplementary Fig. S6. Predicted glycosylation sites in PtFLA6 protein.

Supplementary Data

## Abbreviations:

AGP: arabinogalactan protein
BSA: bovine serum albumin
FLA: fasciclin-like AGP
GFP: green fluorescent protein
GPI: glycosylphosphatidylinositol
GST: glutathione *S*-transferase
qRT-PCR: quantitative real-time RT-PCR
SD: standard deviation
SP: signal peptide
TEM: transmission electron microscopy
YFP: yellow fluorescent protein
WT: wild type.
