# Supplementary material for: Antisense expression of the fasciclin-like arabinogalactan protein FLA6 gene in Populus inhibits expression of its homologous genes and alters stem biomechanics and cell wall composition in transgenic trees
Source: J Exp Bot. 2014 Nov 26;66(5):1291–302. doi: 10.1093/jxb/eru479 (PMC4339592; doi:10.1093/jxb/eru479)
Supplement: Supplementary Data [file supp_66_5_1291__index.html]

Antisense expression of the fasciclin-like arabinogalactan protein FLA6 gene in Populus inhibits expression of its homologous genes and alters stem biomechanics and cell-wall composition in transgenic trees — Antisense expression of the fasciclin-like arabinogalactan protein FLA6 gene in Populus inhibits expression of its homologous genes and alters stem biomechanics and cell-wall composition in transgenic trees — Supplementary Data 

# Antisense expression of the fasciclin-like arabinogalactan protein *FLA6* gene in *Populus* inhibits expression of its homologous genes and alters stem biomechanics and cell wall composition in transgenic trees

## Supplementary Data

Data files

**Files in this Data Supplement:**

- Supplementary Data - Supplementary Data
