# Supplementary material for: Antisense expression of the fasciclin-like arabinogalactan protein FLA6 gene in Populus inhibits expression of its homologous genes and alters stem biomechanics and cell wall composition in transgenic trees
Source: J Exp Bot. 2014 Nov 26;66(5):1291–302. doi: 10.1093/jxb/eru479 (PMC4339592; doi:10.1093/jxb/eru479)
Supplement: Supplementary Data [file supp_eru479_jexbot131391_file001.pdf]

Title: Antisense expression of the fasciclin-like arabinogalactan protein *PtFLA6* gene in *Populus* inhibits expression of its homologous genes and alters stem biomechanics and cell wall composition in transgenic trees

Authors: Haihai Wang[1], Chunmei Jiang[1], Cuiting Wang[1], Yang Yang[1], Lei Yang[2], Xiaoyan Gao[1] and Hongxia Zhang[1,*]

Supplementary data

Table S1. Primers used in this study.

Table S2. GenBank accession numbers or gene models used in this study.

Figure S1. DNA sequence alignment of *PtFLA1-10* from *Populus trichocarpa*. *PtFLA2* and *PtFLA3* is the same gene. The gene models of *PtFLA1-10* were listed in Table S2. The alignment was generated by ClustalX (1.83) (http://www.clustal.org/clustal2/).

Figure S2. Phylogenetic analysis of DNA sequences of *PtFLAs* and *AtFLAs*. *AtFLAs* were classified into group A to D (Johnson *et al.*, 2003), which were distinguished by the black lines. *PtFLA1-10* were clustered into the same subclade as *AtFLA11/12* in group A. The unrooted cladogram was generated with TreeView (version 1.6.6) (http://taxonomy.zoology.gla.ac.uk/rod/treeview.html).

Figure S3. qRT-PCR analysis of the other nine *PtFLAs*. cDNA derived

from different tissues was used for qRT-PCR with gene specific primers (Supplementary Table S1). The experiment was performed three times using different batches of plants. Error bars represent the standard deviation (SD) of three technical replicates using pooled samples of three independent plants. The elongation factor gene *PtEF1β* was used as a reference for normalization. AP, apex; ML, mature leaf; MPe, mature leaf petiole; R, root; UP, upper stem phloem; UX, upper stem xylem; MP, middle stem phloem; MX, middle stem xylem; BP, basal stem phloem; BX, basal stem xylem.

Figure S4. Molecular confirmation of transgenic plants. (A) Schematic map of the *PtFLA6*-antisense construct. LB, Left border; *PtFLA6-Pro*, *PtFLA6* promoter; RB, right border. (B) GUS staining of WT and randomly selected transgenic lines L1, L4, L5, L7, L11, L12 and L14). (C) PCR analysis using the forward primer landing on the *PtFLA6* promoter and the reverse primer landing on the antisense cDNA sequence of *PtFLA6*. Genomic DNA was extracted from leaves of WT and transgenic plants. M, λ-*Eco*T14 I digest DNA markers; P, plasmid vector; W, double distilled water; WT, wild type; L1-L14, different transgenic lines. (C) RT-PCR analysis of *PtFLA6* transcripts in the stems of transgenic plants.

Figure S5. Phenotypic analysis. Plant height (A) and stem diameter (B) of WT and transgenic lines L4 and L14 grown in greenhouse were compared. Error bars represent the SDs from ten plants.

Figure S6. Predicted glycosylation sites in PtFLA6 protein. The N-Glyc sites in PtFLA6 were analyzed in the networks of NetNGlyc 1.0 Server (http://www.cbs.dtu.dk/services/NetNGlyc/). Asparagines predicted to be N-glycosylated were highlighted in red and Asn-Xaa-Ser/Thr sequons in the sequence output below were highlighted in blue. The O-Glyc site prediction was performed in the network of NetOGlyc 4.0 Server (http://www.cbs.dtu.dk/services/NetOGlyc/). The aminos predicted to be O-glycosylated were framed in black boxes.

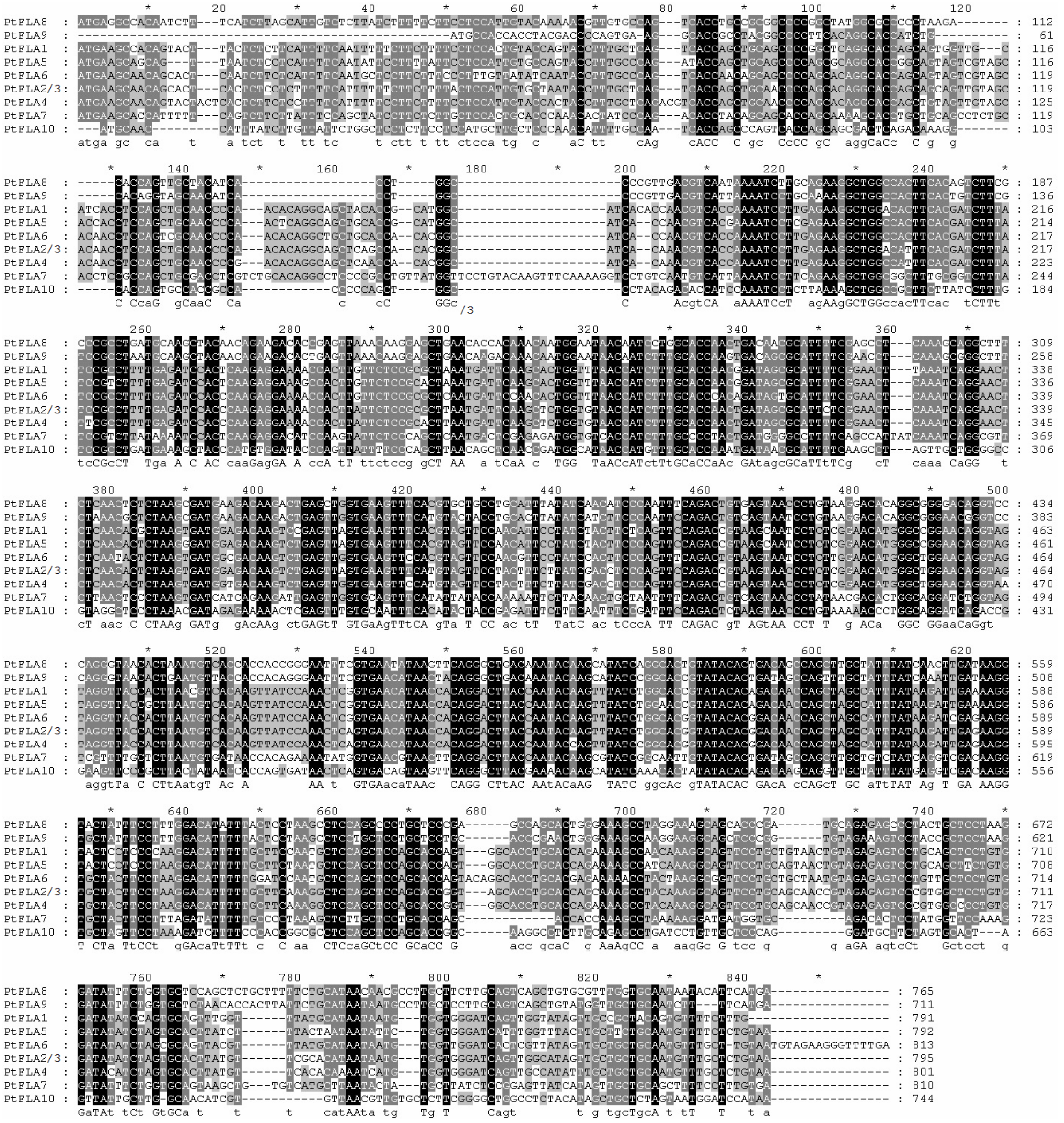

Figure S1. DNA sequence alignment of *PtFLA1-10* from *Populus trichocarpa*. *PtFLA2* and *PtFLA3* is the same gene. The gene models of *PtFLA1-10* were listed in Table S2. The alignment was generated by ClustalX (1.83) (http://www.clustal.org/clustal2/).

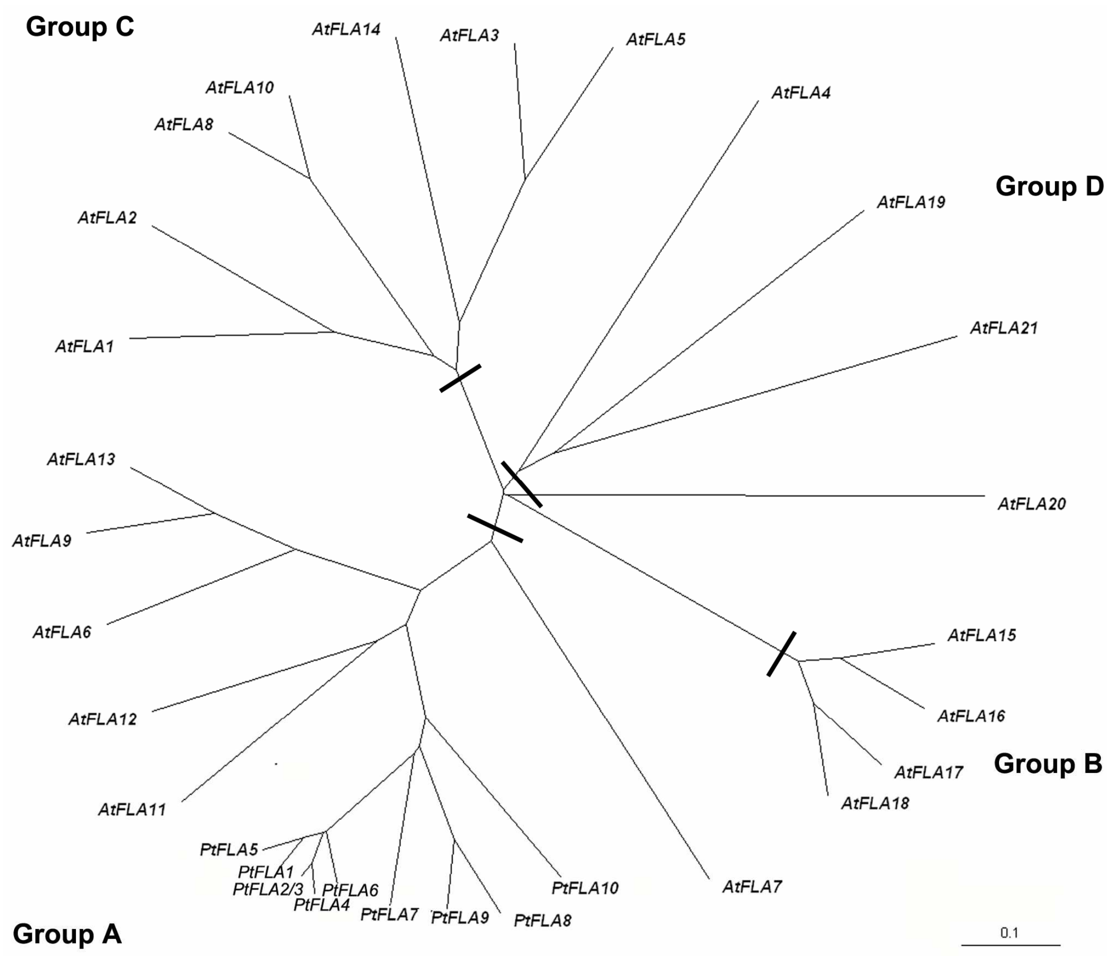

Figure S2. Phylogenetic analysis of DNA sequences of *PtFLAs* and *AtFLAs*. *AtFLAs* were classified into group A to D (Johnson *et al*., 2003), which were distinguished by the black lines. *PtFLA1-10* were clustered into the same subclade as *AtFLA11/12* in group A. The unrooted cladogram was generated with TreeView (version 1.6.6) (http://taxonomy.zoology.gla.ac.uk/rod/treeview.html)..

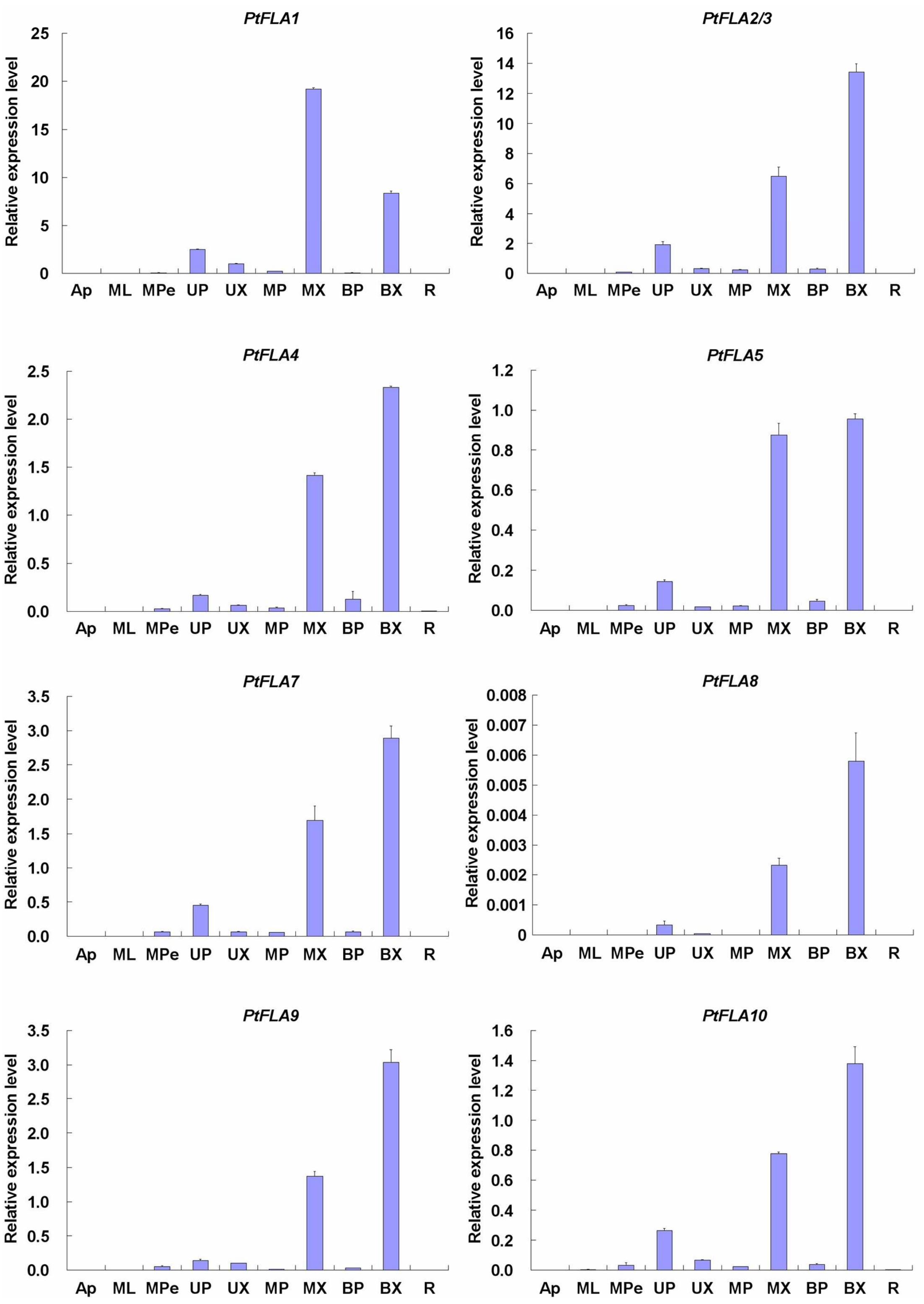

Figure S3. qRT-PCR analysis of the other nine *PtFLAs*. cDNA derived from different tissues was used for qRT-PCR with gene specific primers (Supplementary Table S1). The experiment was performed three times

using different batches of plants. Error bars represent the standard deviation (SD) of three technical replicates using pooled samples of three independent plants. The elongation factor gene *PtEF1β* was used as a reference for normalization. AP, apex; ML, mature leaf; MPe, mature leaf petiole; R, root; UP, upper stem phloem; UX, upper stem xylem; MP, middle stem phloem; MX, middle stem xylem; BP, basal stem phloem; BX, basal stem xylem.

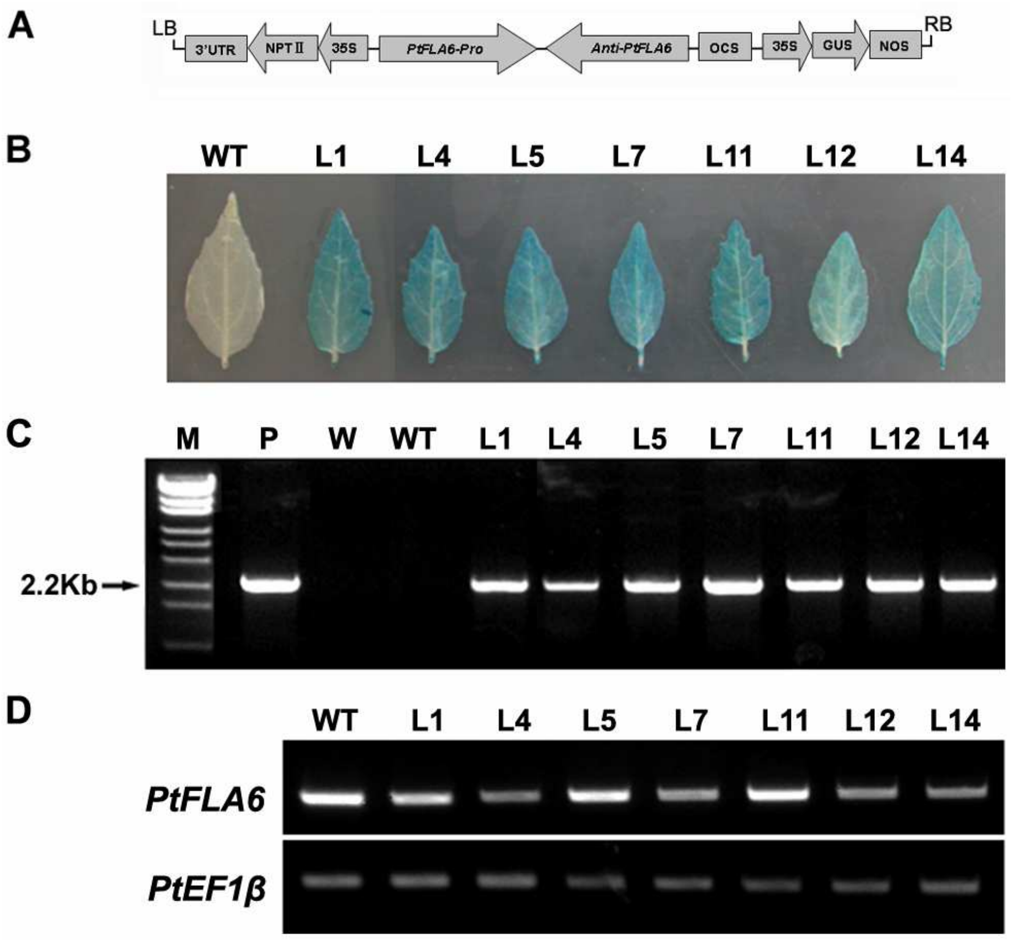

Figure S4. Molecular confirmation of transgenic plants. (A) Schematic map of the *PtFLA6*-antisense construct. LB, Left border; *PtFLA6-Pro*, *PtFLA6* promoter; RB, right border. (B) GUS staining of WT and randomly selected transgenic lines L1, L4, L5, L7, L11, L12 and L14). (C) PCR analysis using the forward primer landing on the *PtFLA6* promoter and the reverse primer landing on the antisense cDNA sequence of *PtFLA6*. Genomic DNA was extracted from leaves of WT and transgenic plants. M, λ-*Eco*T14 I digest DNA markers; P, plasmid vector; W, double distilled water; WT, wild type; L1-L14, different transgenic lines. (C) RT-PCR analysis of *PtFLA6* transcripts in the stems of transgenic plants.

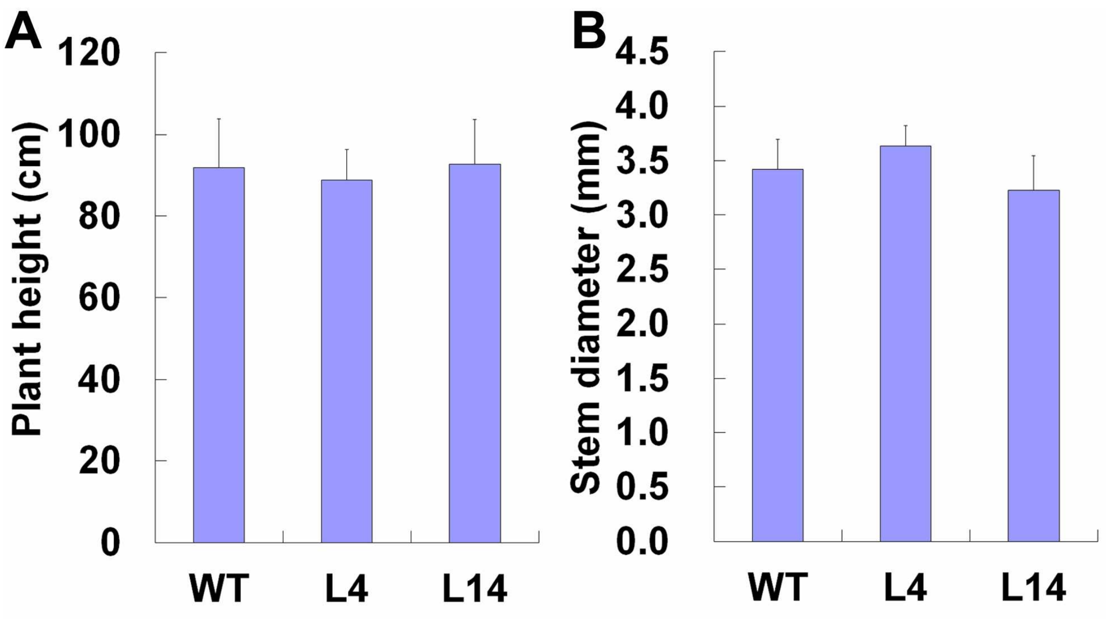

Figure S5. Phenotypic analysis. Plant height (A) and stem diameter (B) of WT and transgenic lines L4 and L14 grown in greenhouse were compared. Error bars represent the SDs from ten plants.

```
>PtFLA6
MKQQHSIFSFSMLLLSLCYINTFAQSPTAAPAQAPAVVVAQPPVATPTQAAAPHGITNVTKILEKAGHFTIFIRLLRSTQ   80
EENHLFSALNDSNTGLTIFAPTDSAFSELKSGTLNTLSDGDKSELVKFHVVPTFLSTSQFQTVSNPLGTWAGTGSRLPLN 160
VTSYPNSVNITTGLTNTSLSGTVYTDNQLAIYKIEKVLLPKDIFGSNAPAPAPVQAPAREKPTKAVPAANVESPVAPVDI 240
SSAVTFMHNNVVVGSLVIVAAAMFACNVEGF                                                  320
```

Figure S6. Predicted glycosylation sites in PtFLA6 protein. The N-Glyc sites in PtFLA6 were analyzed in the networks of NetNGlyc 1.0 Server (http://www.cbs.dtu.dk/services/NetNGlyc/). Asparagines predicted to be N-glycosylated were highlighted in red and Asn-Xaa-Ser/Thr sequons in the sequence output below were highlighted in blue. The O-Glyc site prediction was performed in the network of NetOGlyc 4.0 Server (http://www.cbs.dtu.dk/services/NetOGlyc/). The aminos predicted to be O-glycosylated were framed in black boxes.

Table S1. Primers used in this study.

Primer name	Sequence of primer
PtFLA6-OF	5'-ATGAAGCAACAGCACTCAATCTT-3'
PtFLA6-OR	5'-TCAAAACCCTTCTACATTACAAGC-3'
PtFLA6-PF	5'-GGAATTCAGAGAGAAAGGAAAAGGAGGTTG-3'
PtFLA6-PR	5'-GCGTCGACGGATAAAATGCTCGAAACAATTC-3'
PtFLA1-RF	5'-ATGAAGCCACAGTACTTACTCTCTT-3'
PtFLA1-RR	5'-TGGTTAAACCAGTGCTTGAATC- 3'
PtFLA2-RF	5'- ACCAATACAAGTTTATCTGGCACG-3'
PtFLA2-RR	5'-CAAAACCAGACCAAACCCCAC- 3'
PtFLA4-RF	5'-TCGAGCTTAATTTGCATTGGTATTT-3'
PtFLA4-RR	5'-GGGTGGATCTCAAAAGGCGA- 3'
PtFLA5-RF	5'-CAACTTGCATTGGCATTTCTGA-3'
PtFLA5-RR	5'-TGCTTGAATCATTTAGTGCGGAG- 3'
PtFLA6-RF	5'-GAATTGTTTCGAGCATTTTATCCC-3'
PtFLA6-RR	5'-CTGGAGGTTGTGCTACGACTACTG- 3'
PtFLA7-RF	5'-GATGAAGCACCATTTTTCAGTCTTC-3'
PtFLA7-RR	5'-CAGGACCTTTTGAAACTTGTACAGG- 3'
PtFLA8-RF	5'-TCTGCATAACAACGCCTTGC-3'
PtFLA8-RR	5'-ATGGGAATGACAGGAAAACGTC- 3'
PtFLA9-RF	5'-GTAATGCCACCACCTACGACCC-3'
PtFLA9-RR	5'-CGTTTAGAAAGCCCGCTTTGAG- 3'

PtFLA10-RF	5'-AGGTCGACAAGGTGCTAGTTCCT-3'
PtFLA10-RR	5'-CATTTGAGTCATTGGCCCCACT- 3'
PtEF1β-RTF	5'-GACAAGAAGGCAGCGGAGGAGAG-3'
PtEF1β-RTR	5'-CAATGAGGGAATCCACTGACACAAG-3'
PtCesA4-F	5'-CTGGATCCTTGACCAGTTCCCAAA-3'
PtCesA4-R	5'-ATTAGGCTCACCCTCACGCTCAAA-3'
PtCesA7-F	5'-CTTCCATGTGCACCTTTGAAGCCA-3'
PtCesA7-R	5'-TCAGGAGCTCGAGGTTCTATGCTA-3'
PtCesA8-F	5'-TGACCCACTGAAAGAGCCTCCATT-3'
PtCesA8-R	5'-GTAAGCATGGCAGCACCATCATCA-3'
PtCesA17-F	5'-CTCTAGTCACGGGCAACACACTTT-3'
PtCesA17-R	5'-GTGCACATTGAAGCACCATCGTCA-3'
PtCesA18-F	5'-ACCGCCATTGATCACTGCCAATAC-3'
PtCesA18-R	5'-AGCTGCACCATCATCAGACACGTA-3'
PtrC4H2-F	5'-GAAATGTGCAATTGATCATATTTTG-3'
PtrC4H2-R	5'-ATTGCAGCAACATTGATGTTCTCC-3'
PtrCCoAOMT1-F	5'-CAGTAATTCAGAAAGCTGGTGTTGC-3'
PtrCCoAOMT1-R	5'-GCATCCACAAAGATGAAATCAAAAC-3'
PtrCOMT2-F	5'-TCTTGAAGAATTGCTATGACGCCT-3'
PtrCOMT2-R	5'-GAATGCACTCAACAAGTATCACCTTG-3'

Table S2. GenBank accession numbers or gene models used in this study.

Genes in this study	GenBank accession number/ gene models
PtCesA4	estExt_fgenesh1_pg_v1.C_LG_II0056
PtCesA7	estExt_Genewise1_v1.C_LG_VI2190
PtCesA8	eugene3.00110696
PtCesA17	eugene3.00180941
PtCesA18	eugene3.00040363
PtrC4H2	EU603302
PtrCCoAOMT1	EU603307
PtrCOMT2	EU603317)
PtEF1β	eugene3.00091463
PtFLA1	eugene3.00191024
PtFLA2/3	eugene3.00131210
PtFLA4	eugene3.00130132
PtFLA5	grail3.0094006801
PtFLA6	eugene3.00131208
PtFLA7	grail3.0031016901
PtFLA8	estExt_Genewise1_v1.C_LG_IX4802
PtFLA9	eugene3.00660250
PtFLA10	eugene3.00091518
